# Supplementary material for: Chromothripsis during telomere crisis is independent of NHEJ, and consistent with a replicative origin
Source: Genome Res. 2019 May;29(5):737–49. doi: 10.1101/gr.240705.118 (PMC6499312; doi:10.1101/gr.240705.118)
Supplement: Supplemental Material [file supp_gr.240705.118_Supplemental_file_1.zip › contigs/annotated_contigs/DB41/contig.4.DB41_length_1479_mean_cov_8.63421230561.docx]

**DB41_length_1479_mean_cov_8.63421230561**

```
TAAAAAATAACATATTCTAAAACATAGATTTGGAT|AAAGCATACAAATAAACAAAAAA|AAAAAAAAA|CTAATCAAATTATTTTCAA
                                    >chrX:148654870-148654902 - E=4e-01 p=8e-03 >chr9:
TGGGGCTACAAAGCATTGCATTGTAACCTGAAATCCAAAGCTTACATTGGCTTGAAAATTTGACCAGGCTTACTTAGCTGAAAGAGTCC
24002741-24002956 + E=4e-117 p=1e-02
TTTTAGACTATAAACTTTTGCTAATTTTGTACCAGAAAAACTGGGTTCAAAGATTTTATTCTTCATTCTCCTTTAAATCATATATATAT

ATATATA|TA|CTTAATAATAATAATGAAATTTCTTATGTTAAATAGTCTACAATTCCAAGTTCGTAGTGCCAAGGACTGTCATCAGCA
        >chr3:113480804-113481610 - E=0e+00
AACTCTCTTCCTGAATCCTGAACCTTCATGGTGTATTAGCACTGCAATGACTGGCTTTTGGATAGGCTTTTGTCAGATAAACTGCCTAT

GAAATGACTTTACCATTTTTTTTTTTTGAGATGGAGTTTTGCTCTTGTTGCCTAGGATCGACTGCAATGGCGCAATCTCGGCTCACTGC

AACCTCCACGTCCTGGGTTCATTTGATTCTCTTGCCTCAGCCTCCCGAGTAGCTGGGATTACAGGTATGCGCCACCACGCCCAGCTAAT

TTTGTATTTTTAGTGGAGCCAGGGTTTCTCCAAGTTGGCCAGGCTTGTCTCGAACTCCTGACCTCAGGTGATCCGCCCGCTTTGGCCTC

CCAAAGTGCTGGGATTACAGGCGTGAGCTGCTGTGCCCAGCCAACTTTACCAATTTTTTTCAAAATTCAGGCAATCATGCAGGAAAATT

ATCACCTTCAAAAGAAGTCAAACTAGAAACTGTATGACTACTTTAAAAAGGCAATTGCTTAAAATACTTAGAAACTTTTTTTTTTTGAG

ACAGAGTTTTGCTCTTGTTGCCCAGGCTGCAGTGCAATGGCGCGATCTTGGCTCACCACAACCTCTGCCTCCCGGGTTCAAGCAATTCT

CCTGCTTCAGCCTCCAGAGTAGCTGCGATTACAGGCATGTGCCTCCATGCCCAGCTAATTATGTATTTTTAGTAGAGTTGGGGTTTCTA

CATGTTGGTCAGGC|AACATATGCAAAG|TATATATATATATGATTTTAAAATATTTTTTAAAAGAGTAATTCAGAAAAAGGATAAGGA
                            >chr9:24002968-24003357 + E=1e-221
AACTCAATTGTCCTGATCACGTAAGCAAAACTACTTTGAGATTAGCGTTTAGAACCACCCTTTTTCCCTCAATCTGGTTCCTAGTTCAG

GAAAGGTCATTTTATATACCAGAATAAATGAAAAAAATTATTTCTGCCCTACTGCATTGGACTAGTGTCCTCACTAGGAGTTCATGAAA

CAGTTTCAATAAAAATGCAATTACAATAAGGTATTTTAAATCATATTAATATGATTACCTTAGACTCACATTGAAAATACAGAGAATGT

TTAATTTTTAGAAAATGTTAAACACTTTAGTTTAGTAAATCCTATCATGTTTTCCAGTAATG
```
